# Asian expert recommendation on management of skin and mucosal effects of radiation, with or without the addition of cetuximab or chemotherapy, in treatment of head and neck squamous cell carcinoma

**DOI:** 10.1186/s12885-016-2073-z

**Published:** 2016-01-27

**Authors:** Guopei Zhu, Jin-Ching Lin, Sung-Bae Kim, Jacques Bernier, Jai Prakash Agarwal, Jan B. Vermorken, Dang Huy Quoc Thinh, Hoi-Ching Cheng, Hwan Jung Yun, Imjai Chitapanarux, Prasert Lertsanguansinchai, Vijay Anand Reddy, Xia He

**Affiliations:** Fudan University Cancer Hospital, Shanghai, China; Taichung Veterans General Hospital, Taichung, Taiwan; Asan Medical Center, Seoul, Korea; Swiss Genolier Medical Network, Genolier, Switzerland; Tata Memorial Hospital, Dr. E Borges Road, Parel, Mumbai, 400 012 India; University of Antwerp (UA) and Antwerp University Hospital (UZA), Edegem, Belgium; HCMC Oncology Hospital, Ho Chi Minh City, Vietnam; Queen Elizabeth Hospital, Hong Kong, China; Chungnam National University Hospital, Daejeon, Korea; Chiang Mai University, Chiang Mai, Thailand; Wattanosoth Hospital, Bangkok, Thailand; Apollo Cancer Hospital, Hyderabad, India; Jiangsu Cancer Hospital, Nanjing, China

**Keywords:** Skin and mucosal effects, Radiation, Cetuximab, Chemotherapy, Head and neck squamous cell carcinoma, Recommendations

## Abstract

With increasing numbers of patients with unresectable locoregionally advanced (LA) head and neck squamous cell carcinoma (HNSCC) receiving cetuximab/radiotherapy (RT), several guidelines on the early detection and management of skin-related toxicities have been developed. Considering the existing management guidelines for these treatment-induced conditions, clinical applicability and standardization of grading methods has remained a cause of concern globally, particularly in Asian countries. In this study, we attempted to collate the literature and clinical experience across Asian countries to compile a practical and implementable set of recommendations for Asian oncologists to manage skin- and mucosa-related toxicities arising from different types of radiation, with or without the addition of cetuximab or chemotherapy. In December 2013, an international panel of experts in the field of head and neck cancer management assembled for an Asia–Pacific head and neck cancer expert panel meeting in China. The compilation of discussion outcomes of this meeting and literature data ultimately led to the development of a set of recommendations for physicians with regards to the approach and management of dermatological conditions arising from RT, chemotherapy/RT and cetuximab/RT, and similarly for the approach and management of mucositis resulting from RT, with or without the addition of chemotherapy or cetuximab. These recommendations helped to adapt guidelines published in the literature or text books into bedside practice, and may also serve as a starting point for developing individual institutional side-effect management protocols with adequate training and education.

## Background

**Fig. 1:**
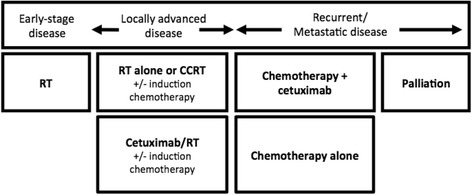
Main nonsurgical treatment modalities for HNSCC based on literature and clinical practice. RT, radiotherapy; CCRT, concurrent chemoradiotherapy; CT, chemotherapy

Head and neck carcinomas account for 5 % of all cancers, and over 90 % are head and neck squamous cell carcinoma (HNSCC) [1, 2]. The landscape of HNSCC treatment has evolved over the past decade. Multiple factors feed into treatment decisions, and a multidisciplinary team approach is important for making treatment decisions. Historically, the standard nonsurgical treatment for locoregionally advanced (LA) disease was radiotherapy (RT) alone, which still is the standard treatment in some parts of Asia along with cisplatin-based concurrent chemoradiotherapy. Cetuximab, an anti-epidermal growth factor receptor (EGFR) monoclonal antibody, was shown to improve loco-regional control rates and survival in combination with RT versus RT alone [3]. Cetuximab plus RT, therefore, further helped to provide an alternative treatment option in the LA-HNSCC population. Based on supporting literature and clinical practice, the main treatment modalities for HNSCC are summarized in Fig. 1.

Epidemiological studies show an increasing incidence of human papillomavirus (HPV)-associated oropharyngeal cancer. HPV-associated HNSCC is recognized as a special entity; patients with such tumours are often younger and have better prognosis, therefore long-term toxicities of therapy are a major issue [4]. Not only in such patients [5], but in the overall management of LA-HNSCC, reduction of treatment-related toxicities is generating more attention, particularly where patient quality-of-life is prioritised as part of the multidisciplinary treatment approach. Concurrent chemoradiotherapy (CCRT) with high-dose cisplatin is known to cause considerable early [6] and late [7] toxicities in HNSCC cases, and that is even the case when using weekly low-dose schedules [8, 9]. The aforementioned Bonner trial,3 comparing cetuximab/RT to RT alone in LA-HNSCC patients, showed superiority of the cetuximab/RT arm with respect to loco-regional control (at 3 years, 47 % versus 34 %) and overall survival (at 5 years, 46 % versus 36 %) after a median follow-up of 54 months. An interesting finding of that study was the remarkable compliance to the cetuximab/RT treatment, with an adherence rate of 90 % [10]. With the exception of acneiform eruptions and infusion reactions, the incidence of grade 3 or greater toxic effects, including mucositis, did not differ significantly between the two arms of the study. A better compliance with cetuximab/RT than with cisplatin-based CCRT was also observed in a direct comparison of both approaches after cisplatin-based induction chemotherapy (ICT) in the TREMPLIN study, a larynx preservation study in patients with larynx and hypopharynx cancer who were candidates for total laryngectomy [11]. Interestingly, the better compliance was observed despite the fact that a higher incidence of grade 3 in-field skin toxicity was observed. Japanese oncologists also used an opioid-based pain control program more systematically to improve compliance with CRT in head and neck cancer patients [12].

With an increasing number of patients with unresectable LA-HNSCC receiving cetuximab/RT, several guidelines on the early detection and management of skin-related toxicities have been developed, which address pathogenesis, pathophysiology and clinical aspects in patients experiencing these side effects [13, 14]. At the same time, as mentioned by several oncologists, the reported rates of skin toxicity and mucositis with cetuximab/RT in daily practice may be higher than that reported in the pivotal studies with this combination [15, 16]. Given the existing management guidelines for these treatment-emergent conditions, clinical applicability and standardization of the grading methods has remained a cause of concern globally, particularly in Asian countries, because of racial and ethnic variations in tumour subsites, causative factors, skin conditions, hospital radiotherapy set-ups, patient management protocols and so on. Notwithstanding the fact that, thus far, no robust data can be found in the literature in favour of a link between ethnic differences and variations in skin sensitivity to cetuximab; such a relationship might explain the higher incidence and severity of cutaneous reactions observed consistently in the Asian population compared with Western patient cohorts. Therefore, this study was developed in an attempt to compile literature and clinical experience from across Asian countries, to determine a practical and implementable set of recommendations for Asian oncologists to manage skin- and mucosa-related toxicities caused by different types of radiation, with or without the addition of cetuximab or chemotherapy.

## Methods

In December 2013, an international panel of experts in the field of head and neck cancer management convened for an Asia–Pacific head and neck cancer expert panel meeting in China. The panel comprised members who are experts in the fields of head and neck cancer medical oncology and radiation oncology. As pre-meeting preparation, the panel members participated in a pre-meeting survey to assess the occurrence of skin and mucosal toxicities observed with cetuximab/RT treatment, along with the management practices followed in their respective practice, institute or hospital. These pre-meeting survey results were used as the basis for the expert panel discussion, which ultimately led to the development of a set of recommendations for physicians with regards to the following:

- Approach to and management of dermatological conditions arising from RT, CCRT and cetuximab/RT).
- Approach to and management of mucositis resulting from radiation, with or without the addition of chemotherapy or cetuximab.

During this whole process, it was kept in mind that treatment strategies are changing over time and that survivorship issues are becoming more prominent. Reducing late toxicities is thereby of crucial importance.

### Radiation dermatitis and skin toxicity from cetuximab/RT

#### Literature review and clinical experience

Anti-EGFR treatment outcomes in a variety of solid cancers, including HNSCC, correlate with the degree of skin rash [17]. The acneiform skin eruptions observed with cetuximab may be better described as “folliculitis” because of its pathophysiology and distribution areas. Overall, skin rashes are manageable and reversible [18]. In the Phase II TREMPLIN study, the cetuximab/RT arm showed a higher number of patients with grade 3–4 in-field skin toxicity than the cisplatin-based CCRT arm. However, not only the occurrence of the in-field dermatitis differs, but also the type of in-field skin toxicity. There are both pathophysiological and clinical differences in the dermatitis induced by RT alone, CCRT and cetuximab/RT (Table 1) [18].

**Table 1:** Pathophysiological and clinical differences in radiation dermatitis with RT/CRT and cetuximab + RT

RT/CRT alone	Cetuximab + RT
Pathophysiological (for more details, please refer to text)	
	
Clinical	
Onset of dermatitis is within 3–5+ weeks of treatment	Onset of dermatitis is within 1 or 2 weeks of treatment
No crusting	Crusting is present, which can result in sustained microtrauma, bleeding, and discomfort and can lead to infection

# Images courtesy of Dr. Merlano

Distinguishing characteristics of cetuximab/RT-associated dermatitis consist of marked xerosis, an intense inflammatory response in the sub-epidermis (indicating an immunological- and cytokine-mediated response at the level of the epidermis and dermis), and the inhibition of anti-microbial peptides, which increases the risk of a superinfection. There may be loss of continuity of the epidermis, leading to exudation of fluids and formation of crusts. These crusts are comprised of inflammatory exudate and exfoliated corneocytes; they compromise the healing of the affected area, and are susceptible to sustained microtrauma and are thereby prone to abrasion, bleeding, discomfort and/or pain and risk of superinfection. Contrary to what is observed with cetuximab/RT, crusting is typically absent with radiation alone or with CCRT. With CCRT, the dermatitis is associated with a dry desquamation and exfoliated corneocytes, occurring before moist desquamation and exposure of the underlying dermis. With higher dosages of radiation, as seen with modern and novel methods of irradiation, skin necrosis and ulceration of dermis may be noted frequently. The cetuximab/RT-associated dermatitis appears to be more severe than that with RT alone or CCRT, and has an earlier onset at around 1–2 weeks of starting treatment. However, it also resolves more rapidly, approximately 1–2 weeks after the completion of treatment (clinical practice).

There is a need to follow a different grading system for radiation dermatitis, to distinguish that which arises from cetuximab/RT and that which occurs with RT alone. The new grading system and management guidelines published in *Annals of Oncology* help to understand, assess, evaluate and manage cetuximab/RT-induced radiation dermatitis more successfully [19]. While there is currently no validated, standardized, uniform method of grading, thus preventing the development of radiation dermatitis, intervention at an early stage is crucial for effective management.

In general, patients with grade 1–3 reactions can be managed as outpatients, although this should be decided on an individual patient basis. Initially, patients must be monitored weekly by the management team for signs of early skin reactions (for the first 2 weeks), until the first sign of erythema, at which point monitoring should be more frequent (at least twice weekly) and intense. Patients developing severe early erythema should be monitored closely throughout treatment. Bypassing early monitoring of dermatitis can eventually lead to abrupt discontinuation of therapy, thereby jeopardizing a beneficial outcome of the treatment. Continuation of cetuximab treatment depends on the grade of radiation dermatitis observed. In cases of grade 3 dermatitis, it may be appropriate to consider a brief interruption for 4–5 days in the treatment of severe grade 3 dermatitis, especially with suspected superinfection or with a radiation doses as low as 50 Gy (or a cumulative dosage reaching a total of 50 Gy). Cetuximab can be restarted as soon as the severity of dermatitis reduces to grade 2. While grade 4 dermatitis is considered to be a rare event, cetuximab, and/or other systemic anticancer treatments, should be discontinued.

Overall, patients should be provided with written information on how to manage their skin reactions, and the use of a nursing diary for the same purpose is recommended. Management of dermatitis can be categorized under general and grade-specific management (Table 2) [18]. An expert team, comprising of a dermatologist and nursing care, is crucial in symptomatic and supportive care to adequately monitor and manage radiation dermatitis.

**Table 2:** Radiation dermatitis: grading and general management recommendations

Grade of radiation dermatitis	Grade 1	Grade 2	Grade 3	Grade 4
Definition of radiation dermatitis (NCI CTCAE, v3.0)	Faint erythema or dry desquamation	Moderate to brisk erythema; patchy, moist desquamation, mostly confined to skin folds and creases; moderate oedema	Moist desquamation other than skin folds and creases; bleeding induced by minor trauma or abrasion	Skin necrosis or ulceration of full thickness of dermis; spontaneous bleeding from involved site
General management approaches	See General management			
	Maintain hygiene and gently clean and dry skin in the radiation field shortly before radiotherapy			
	Topical moisturisers, gels, emulsions and dressings should not be applied shortly before radiation treatment as they can cause a bolus effect, thereby artificially increasing the radiation dose to the epidermis			
Grade-specific management approaches	Use of a moisturiser is optional	Keep the irradiated area clean, even when ulcerated		Verify that radiation dose and distribution are correct
	If anti-infective measures are desired, antibacterial moisturisers (e.g. triclosan or chlorhexidine-based cream) may be used occasionally	In the absence of clinical signs of infection, one or combinations of the following topical approaches may be used:		Requires specialised wound care with the assistance of the radiation oncologist, dermatologist and nurse, and should be treated on a case by case basis
		•- Drying gels, possibly with the addition of antiseptics (e.g. chlorhexidine-based creams)		
		•- An anti-inflammatory emulsion, such as trolamine		
		•- Hyaluronic acid cream		
		•- Hydrophilic dressings, applied after radiotherapy to the cleaned, irradiated area, which may provide symptomatic relief		
		•- Zinc oxide paste, if easy to remove prior to radiotherapy		
		•- When used, silver sulfadiazine or beta glucan cream should be applied after radiotherapy (possibly in the evening) after cleaning the irradiated area		
		•- Where infection is suspected:		
		•- The treating physician should use best clinical judgement for identifying infection, including the consideration of swabbing the area for identification of the infectious agent		
		•- Topical antibiotics (should not be used prophylactically)		
		•- Doxycycline is not recommended at this stage		
		•- Blood granulocyte counts should be checked, particularly if the patient is receiving concomitant chemotherapy		
		•- Blood cultures should be carried out if there are additional signs of sepsis and/or fever		
Management team	Can be managed primarily by nursing staff	Can be managed by an integrated management team comprising the radiation oncologist, nurse, medical oncologist (where appropriate) and dermatologist, as required	Should be managed primarily by a wound specialist, with the assistance of the radiation oncologist, medical oncologist (where appropriate), dermatologist and nurse, as required	
		Skin reactions should be assessed at least once a week		

General management of radiation dermatitis, as mentioned in Table 2, includes [18] skin hygiene (washing no more than twice a day with pH 5 soap and clean towels); shaving to reduce folliculitis risk; transparent dressings to allow monitoring for infection; debridement to reduce superinfection risk; monitoring for systemic inflammation; and avoidance of aloe vera, scratching, local trauma, exposure to sunlight and dressings that might be responsible for deviations from treatment protocols in terms of radiation dose reduction. According to Japanese experience, radiation dermatitis can be manageable by gentle washing and moistening of the wound-healing environment [20].

The panel found deficiencies in the management of radiation dermatitis that still remain to be addressed, including the following: inconsistent toxicity criteria; subjective grading of reactions that impedes the interpretation of toxicity findings; little evidence to indicate that any of the currently available products can prevent the development of these skin reactions; and insufficient understanding of the biological mechanisms responsible for the skin toxicity of individual agents, as a greater understanding would lead to the development of rational and more effective management strategies for the skin reactions of patients receiving cetuximab/RT.

## Results

### Recommendations based on clinical practice

The recommendations are based on prevention, early warning signals, management of radiation dermatitis and dose adjustment for cetuximab and radiation. In clinical practice, although the overall reporting of grade and severity of radiation dermatitis in patients receiving cetuximab/RT is similar to that reported in the Bonner trial, a certain amount of variation in the grading cannot be denied. This highlights subjective differences including temporal, interpersonal or treatment biases that may be occurring in the assessment of this condition. This needs to be addressed by a standardized and more objective assessment tool.

The group indicated that it is important to assess exactly when the toxicity starts to develop and not only to look for the maximum grade of toxicity. If skin reactions are already seen in the first or second week of therapy, one would expect more toxicity than when skin reactions are observed for the first time in the third or fourth week of treatment. Moreover, factors like temperature (hot summers/winters) may also affect the grading system. Patients may be assessed by different doctors/observers at different times, which may lead to different grading in the same patient. Even if the criteria are listed in the text, perception may differ between different physicians. The subjective nature of assessment may allow for bias as some physicians are cautious or sometimes less experienced, while others may be more experienced when dealing with the same condition.

Based on the above discussions, the group agreed that there is a need for a new objective method of classification/grading system of radiation dermatitis; for example, having a standard image of each grade. A new grading system may be developed in Asian countries, depending upon ethnic variations, based on crusting, infection and interindividual variations such as skin colour. Any images must be obtained under standard conditions for the hospital or country for such assessments and grading. The guidelines for grading of the radiation dermatitis must take into account climatic (i.e. tropical, sub-tropical etc.) and geographical (i.e. altitude, ethnic variations etc.) factors. A multidisciplinary approach should be considered in defining a new clinically assessable grading system in Asia.

### Recommendations for management of skin conditions

The expert panel indicated that prophylactic treatment is important for both the development of skin eruptions and prevention of superinfection. Immunological reaction and superinfection are two important factors to be considered in the treatment of cetuximab/RT-induced radiation dermatitis. Antihistamines and antibiotics can be considered for the same. Inflammatory reaction is critical in the pathophysiology of cetuximab/RT-induced radiation dermatitis. The panel members recommended against empiric use of prophylactic oral antibiotics and oral corticosteroids, however consideration may be given on a case-by-case basis for oral medications to achieve symptom control and prevent further aggravation of the condition.

This decision must be taken based on the clinical assessment and judgement of the physician after consultation with a dermatologist. Maintenance of hygiene and careful cleaning of the skin were considered the best methods for prevention of severe skin toxicities. These measures are especially important in patients who may have certain predispositions that categorize them as high risk for development of severe skin toxicities, such as having a small posture with a relatively short neck, skin folds in the neck, moist sweaty skin, and use of an immobilization mask. Education of both patient and caregiver is of utmost importance in this condition. For prevention, no clear documentation in the literature or practice exists that can be recommended for all cases. Therefore, it is important that a multidisciplinary approach is followed while designing protocols to manage such conditions. Practice guidelines recommended by the Asian experts are summarized in Table 3, based on guidelines listed in Table 2.

**Table 3:** Common clinical practices for management of radiation dermatitis in Asian countries

	Grade 1	Grade 2	Grade 3	Grade 4
Local treatment	• No treatment is required	• Keep the site clean and dry	• Keep the site clean and dry	• Keep the site clean and dry
	• Avoid rubbing and maintain moisture and hygiene	• Topical treatment with antiseptics/antibiotics/steroids is recommended	• Topical treatment with antiseptics/antibiotics/steroids is recommended	• Topical treatment with antiseptics/antibiotics/steroids is recommended
	• Topical treatment with antiseptics/antibiotics/steroids may help			
Systemic treatment	• No treatment is required	• No treatment is required	• Oral antibiotics, pain-killers, corticosteroids or antihistamines for symptom relief	• Oral antibiotics, pain-killers, corticosteroids or antihistamines for symptom relief
	• Regular monitoring is recommended	• Oral antibiotics, pain-killers, corticosteroids or antihistamines for symptom relief		
			• Temporary discontinuation or delay of cetuximab treatment	
				• Temporary discontinuation of cetuximab and radiation treatment

Based on the above discussions, the expert panel recommended some preventive measures that are practiced by almost all of the attending experts:

- Physician and patient education for skin care.
- Maintaining clean and dry skin, and avoiding perspiration during and especially after exposure to radiation dosing; the skin lesion with dermatitis should be kept moist.
- No viscous creams or jellies to be applied within the field of radiation during the radiation phase.
- Close monitoring once a week during start of therapy; and with emergence of erythema, monitoring must be more frequent up to twice a week, with utmost attention to early management strategies of the condition.

The expert panel overall agreed to the radiation dermatitis “management” guidelines laid down in literature (Table 2). Topical steroids may be necessary for grade 2 and 3 toxicity but should not be administered for a long time. The feasibility of its use should be assessed by a multidisciplinary team involving dermatologists at the treating centre. Alternatively, the combination of topical glucocorticosteroids plus local antiseptics/antibiotics might be useful. Doxycycline, as an anti-inflammatory agent with antibiotic properties, is worth considering on a case-by-case basis in prevention as well as in grade 1–2 severity, to prevent further progression to grade 3 or higher.

However, as mentioned earlier, dermatitis resulting from RT alone and that induced by cetuximab plus radiation (in the irradiated field), have different pathophysiological mechanisms. As cited by Russi EG et al. [19], the grading and management of radiation dermatitis is often not applicable to radiation in-field dermatitis as it does not include the associated side effects of cetuximab, and vice versa, the toxicity grading and management of the systemic cetuximab may not be applicable when the reactions are confined to a limited skin surface, as seen in the irradiated field. These issues can explain the different ‘in-field toxic effect’ rates reported in different studies and in clinical practice, also affecting management of the condition. Based on this observation and experience, Russi et al. proposed a grading system and recommendations for the management of skin conditions arising from cetuximab plus radiation in a ‘Letter to Editor’ article published in the *Annals of Oncology* in July 2013.

The expert group recommended that this type of grading system (Table 4) may be more pragmatic in clinical practice and should be considered when managing cases of cetuximab/RT-induced dermatitis.

**Table 4:** Proposal of a new grading system for bio-radiation dermatitis^a^

TERM	G1	G2	G3	G4
Dermatitis Bio-radiation	Faint erythema or dry desquamation; and lesions due to bio-treatment (e.g. xerosis, papules, pustules, and other clinical signs) which may or may not be associated with symptoms of pruritus or tenderness.	Moderate to brisk erythema; patchy moist desquamation in folds and creases; lesions due to bio-treatment (e.g. crusts, papules, pustules, and other clinical signs) mostly confined to less than 50 % of radiated area; bleeding lesions with friction or trauma.	Moist desquamation in areas other than skin folds and creases; extensive (>50 % of involved field) confluent lesions due to bio-treatment (e.g. crusts, papules, pustules, and other clinical signs) associated to bleeding by minor trauma or abrasion.	Life-threatening consequences; skin necrosis or ulceration of full thickness dermis; extensive (>50 % of involved field) confluent lesions due to bio-treatment (e.g. crusts, papules, pustules, and other clinical signs) associated to signs of spontaneous bleeding. Systemic inflammation response syndrome (SIRS)
Activity of Daily living (ADL)	No limiting age-appropriate ADL	Limiting age-appropriate instrumental ADL	Limiting self-care ADL	
Action	Topical therapy indicated (moisturizers, corticosteroids, antibiotics)	Topical and oral therapy indicated	Topical and oral therapy indicated; dressing and wound indicated; inpatient therapy may be necessary	Hospitalize the patient
Grade-specific management approaches	Weekly follow-up is adequate, unless rapid progression is noted	Consider twice-weekly assessments to monitor rapid change	Evaluate the need for daily assessment Closely monitor signs of local or systemic infection For grade 3 reactions occurring at <50 Gy, consider brief interruption in treatment	Consider interrupting treatment with both radiotherapy and cetuximab. Cetuximab should be interrupted until the skin reaction has resolved to at least grade 2 In the case of severe superinfection, consider the use of i.v. antibiotics if unresponsive to oral antibiotics

^a^Adapted from references 18 and 19

**Fig. 2:**
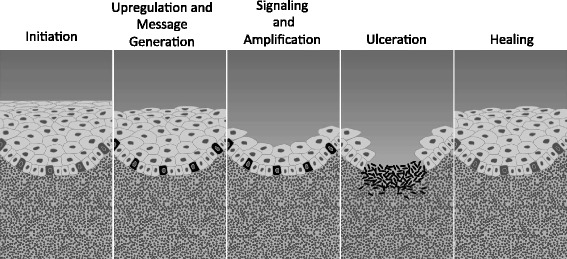
Pathobiology perspective: a multiple mechanism model. # Image courtesy of Keefe and Sonis. NB: The upregulation and message generation phase involves the activation of a number of signalling pathways and transcription factors, most importantly NFκB, which in turn mediates gene expression and synthesis of various inflammatory molecules including proinflammatory cytokines. Signal amplification is the third phase of mucositis development where the inflammation signal is further amplified as a consequence of proinflammatory cytokines, with subsequent further tissue damage as a result of increased apoptosis

The expert panel proposed that the dose reduction scheme for cetuximab-induced > grade 3 skin reactions (mainly acne-like rash occurring outside the radiation field) may also be valid in cetuximab/RT-induced in-field dermatitis (see also Fig. 2). The panel opined that in radiation dermatitis grade 3, cetuximab may be briefly interrupted when occurring at <50 Gy. In grade 4 radiation dermatitis, cetuximab may be omitted until resolution to grade 2. Radiotherapy should only be stopped in cases of grade 4 radiation dermatitis, which is fortunately rarely seen.

Temporary interruption or discontinuation of cetuximab treatment while waiting for ≥ grade 3 radiation dermatitis to resolve to grade 2 does not require a repeat loading dose of cetuximab to be administered in the majority of cases, because this resolution (downgrading), as documented in the guidelines, generally occurs within a week or two of cetuximab dose interruption or discontinuation. However, with good debridement, skin care, hydrocolloid gels, and topical antibiotics, dose delays in cetuximab or radiation may be completely avoided in most cases.

### Mucositis arising from cetuximab/RT

#### Literature review and clinical experience

Between 30 % and 60 % of patients receiving RT for HNSCC may develop oral mucositis, and greater than 90 % of patients receiving CCRT are affected [21, 22]. The degree and duration of mucositis in patients treated with RT are related to radiation source, cumulative dose, dose intensity, volume of radiated mucosa, smoking, alcohol consumption, and oral hygiene [23, 24].

The exact pathophysiology of mucositis is not completely understood. Principally, it is thought to have two mechanisms: direct mucositis and indirect mucositis, caused by chemotherapy and/or radiation therapy.

1. **Direct mucositis:** The epithelial cells of the oral mucosa undergo rapid turnover, usually every 7 to 14 days, which makes these cells susceptible to the effects of cytotoxic therapy. Both chemotherapy and radiation therapy interfere with cellular mitosis and reduce the ability of the oral mucosa to regenerate [21].
2. **Indirect mucositis:** Oral mucositis can also be caused by the indirect invasion of Gram-negative bacteria and fungal species. Patients are at increased risk of oral infections when they are neutropenic, and this usually happens when indirect stomatotoxicity appears [24].

In the literature, pathogenesis of mucositis has been described in four phases [25]: an inflammatory/vascular phase, an epithelial phase, an ulcerative/bacteriologic phase and a healing phase. The first signs of mucositis are white appearances of the mucosa such as hyperkeratinization and edema of the mucosa and formation of pseudomembranes, and red appearances resulting from hyperemia and epithelial thinning such as vascular damage and endarteritis. With 180–220 cGy radiation per day, mucositis with erythema is noted within 1 to 2 weeks and increases throughout the course of therapy to a maximum in 4 weeks, with persistence until 2 or more weeks after the completion of therapy.

A multiple mechanism model was suggested by Keefe and Sonis [26], which divided the process into five stages: initiation, upregulation and message generation, signalling and amplification, ulceration and healing (Fig. 2).

**Fig. 3:**
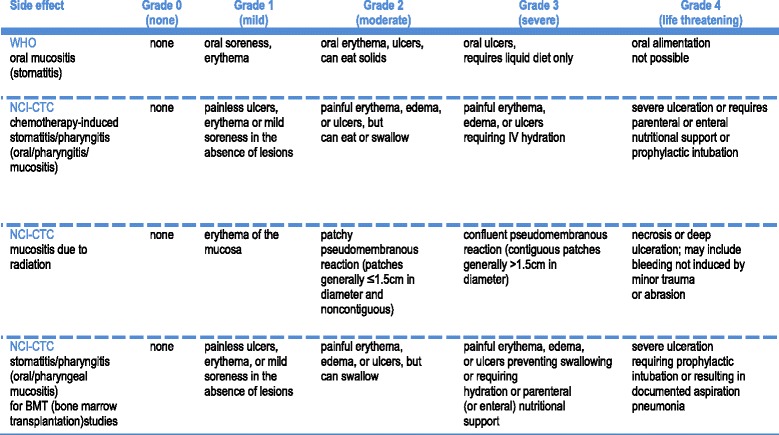
Toxicity grading of oral mucositis according to WHO and NCI-CTC criteria (CTCAE 4.0)

Toxicity grading of oral mucositis according to WHO and NCI-CTC criteria (version 4.0) [27] is shown in Fig. 3. These are commonly-used assessment scales to grade the severity of oral mucositis that might impact negatively on compliance of treatment guidelines in terms of dose intensity. Various risk factors for oral mucositis are chemotherapy dose and protocol, concomitant head and neck RT, microtrauma, pretreatment oral status, and patient factors such as lifestyle and habits. Various differential diagnoses also need to be considered because some conditions including oral thrush, aphthous ulcer, hypovitaminosis, and chronic trauma, such as denture-related trauma, can coexist in immunocompromised patients.

Basic oral care guidelines have been updated for the prevention and treatment of mucositis, including [28]: dental assessment, and care prior to treatment, during treatment and during follow-up; basic oral care including an ultra-soft toothbrush with regular replacement of the toothbrush; bland rinses; promoting mucosal moisturization and protection; and regular check-up for fungal, bacterial or viral infections at follow-ups. For prevention, alternative therapies that can be given include vitamins A, E, and B12, folate, diet supplements, glutamine, aloe vera and PV701, a milk-derived protein extract. Management of oral mucositis can be systemic and topical, as described in Table 5.

**Table 5:** Management of oral mucositis

Systemic	Topical
1. Pain management	Diluting agents^b^: Saline, bicarbonate rinses, frequent water rinses, dilute hydrogen peroxide rinses
• Analgesics: WHO ladder	Topical anaesthetics^b^: Dyclonine HCl, xylocaine HCl, benzocaine HCl, diphenhydramine HCl
• Adjuncts: Relaxation, imagery, biofeedback, hypnosis and transcutaneous electrical nerve stimulation	
	Analgesic agents^b^: Benzydamine HCl
• Beta-carotene	
	Coating agents^c^: Kaolin-pectin, aluminium chloride, aluminium hydroxide, magnesium hydroxide, hydroxypropyl cellulose, sucralfate
2. Radioprotectors^a^	
	Lip Lubricants^c^: Water-based lubricants, lanolin
• Amifostine: Scavenge free radicals	
3. Biologic Response Modifiers^a^	
• G-CSF, GM-CSF, Keratinocyte Growth Factor	

^a^More relevant in Bone Marrow Transplant cases and not crucial in radiotherapy patients

^b^Most practiced and accepted form of topical therapy

^c^Though mentioned in-frequently in literature and case discussions, they have failed to generate sufficient impact in routine practice

As observed in the Bonner trial [3], the incidence of grade 3–4 mucositis and dysphagia did not differ in the cetuximab/RT arm vs. RT alone, with 55.8 % vs. 51.9 % , and 26 % vs. 29.7 % respectively; while in the TREMPLIN study, the occurrence of grade 3–4 mucositis was 45 % with cetuximab/RT versus 47 % with CCRT. Asian clinical studies in Chinese [29] and Japanese [16, 30] populations have also shown a similar or sometimes worse outcome of cetuximab addition to RT, versus RT alone, upon the occurrence of mucositis in these patients.

Despite that, there is a lack of sufficient literature to differentiate pathophysiological differences between mucositis arising from RT alone, CCRT and cetuximab/RT. Clinically, the nature and distribution of mucositis with cetuximab/RT is found to be similar to that with RT and CCRT. However, in the mucositis observed with cetuximab/RT, it seemed that some mucosal inflammation appeared in non-irradiated areas, but effects from radiation scatter cannot be ruled out in these cases, although this was not found in the Bonner study [3].

Identification of risk factors is one of the crucial aspects related to mucositis. Risk of mucositis has classically been directly associated with modality and intensity of radiation [31, 32]. Clinical perception, though not clearly supported in literature, has indicated that combination therapy, either with cisplatin or with cetuximab with RT, may increase the severity of oral mucositis. Incidence and severity of acute mucosal toxicity has not generally been significantly reduced by utilization of state-of-the-science radiation technologies (for example, volumetric-modulated arc therapy). Genetic polymorphisms or ethnic and racial intrinsic sensitivities may play a role. Patient-related risk factors such as co-morbidities (for example, malnutrition and diabetes) and lifestyle habits (smoking, tobacco chewing, poor oral hygiene, and alcohol) can contribute, and significant salivary hypofunction/xerostomia and/or antiemetic drugs may cause increased discomfort from oral mucositis.

### Discussion and recommendations based on clinical practice

Based on the above discussions, the group of experts proposed to categorize patients at risk of developing severe mucositis, as shown below:

- Patient-related risks: smoking, poor hygiene, clinical co-morbidities (such as diabetes, superadded candidal thrush).
- Tumour-related risks: site-related such as the oropharynx; tumours close to the midline are more related to mucositis than unilateral tumours.
- Treatment-related risks: radiation dose intensity, technique-related.

### Grading of mucositis

Similar to radiation dermatitis, the expert group opined that no single grading system can completely address the grading of mucositis adequately and in a reproducible manner. In the event of one or multiple differential diagnoses co-existing with oral mucositis, the grading becomes highly subjective. Based on the above discussion, and similar to a need for having a standardised grading system for mucositis, the group recommended that a photographic method of assessing the severity of mucositis will be crucial for correlating the corresponding mucositis severity assessment criteria such as those of WHO and NCI-CTC.

### Management of mucositis

#### Prevention

Quoting from literature [33] and institutional experience [34], a considerable amount of debate and varied schools of thoughts exist on the optimal and correct radiation techniques and modalities that truly benefit the patients, spare normal organ function and avoid exposure to unnecessary toxicity.

Although understanding and handling of newer radiation techniques is still being improved and can be mastered effectively with increasing experience, there is more mucositis with newer radiation technology. Mucositis may be more intense with volumetric arc-related technique/IMRT as compared with 3D-CRT in certain cases, because of the greater area of radiation exposure and hence increased damage to mucosa, especially in cases of bilateral nodal involvement or bulky primary tumours. When combined with cetuximab, there appears to be more lesions in the mucosa resulting from IMRT in clinical practice. But at the same time, it is also important to note that the potential advantage of saving the critical organs with newer technologies outweigh some of the manageable and transient side effects resulting from them [34, 35].

In clinical practice, parenteral feeding is not encouraged unless there is aspiration or dramatic weight loss of greater than 10 %. Stimulating the patient to swallow naturally during the radiation treatment phase is always useful and also protects the pharyngeal muscles from long-term residual side-effects. Some centres also use nasogastric tubes if required, rather than percutaneous endoscopic gastrostomy (PEG), at an early stage to avoid weight loss and nutritional deficiency from dysphagia.

Incidence of mucositis may be high in patients receiving induction chemotherapy regimens, such as the new standard docetaxel-cisplatin-5-fluorouracil (TPF) regimen, followed by definitive CCRT. [36] Unlike in the Bonner study, wherein almost 70 % of patients had a good performance status, in daily practice cetuximab/RT is often used in patients who are elderly, have a poor performance status or have a contraindication for cisplatin or cannot tolerate it. This may confound the severity of mucositis that is seen in practice to that observed in the Bonner study. The group concluded that for such patients who are relatively frail compared with the better performance status in patients enrolled in studies, but eligible to receive intensive and planned therapy, any form of combination therapy may be more toxic [37, 38]. The group also concluded that for many poor performance patients, radiation alone should be sufficient, and the choice of cetuximab/RT versus CCRT should predominantly be made in patients fit enough to receive CCRT. Cetuximab/RT could further be considered as an option for poor performance patients who despite that are deemed to need a combined approach. Common clinical practices for management of mucositis set by the Asian experts are summarized in Table 6.

**Table 6:** Common clinical practices for management of mucositis in Asian countries.

Grade 1	Grade 2	Grade 3	Grade 4
• Maintain oral hygiene	• Maintain oral hygiene	• Maintain oral hygiene	• Maintain oral hygiene
• Frequent mouthwash use with agents like betadine, sodium bicarbonate	• Frequent mouthwash use with agents like betadine, sodium bicarbonate	• Frequent mouthwash use with agents like betadine, sodium bicarbonate	• Frequent mouthwash use with agents like betadine, sodium bicarbonate
	• Thymol and aspirin gargles/NSAIDs/local anesthetics for pain relief		
			• NSAIDs/opioids/local anesthetics for pain relief
• Thymol and aspirin gargles/ NSAIDs/local anesthetics for pain relief		• NSAIDs/opioids/local anesthetics for pain relief	• Systemic continuous use of steroidal therapy for mucositis prevention/therapy not recommended
			• Parenteral nutrition used only if the bowel is not working or there are serious contra-indications to the placement of a device for enteral nutrition
		• Parenteral nutrition used only if the bowel is not working or there are serious contra-indications to the placement of a device for enteral nutrition	
			• Stop radiation and cetuximab till the condition is resolved
		• Cetuximab dosing may be interrupted for a week or two, till the reaction has resolved to grade 2	

Based on the above discussion, the group made a few recommendations in the prevention of mucositis as a general measure for radiation therapy with or without concurrent systemic treatment, including cetuximab:

- Physician and patient education for mucosal care.
- For prevention of mucositis, all experts recommended to follow the MASCC [28] guidelines in clinical practice. Adding saline and sodium bicarbonate rinses to the prevention guidelines was suggested. It was also mentioned that honey, used in some parts of the world, may be an effective and feasible option for preventing mucositis.
- Maintaining oral hygiene is of utmost importance in preventing mucositis. Frequent mouthwash use is also an important factor.
- Tobacco, betel nut-chewing, smoking etc. adds to irritability and hence should be avoided as a precautionary measure.
- Use of midline radiation blocks and three-dimensional radiation treatment to reduce mucosal injury is recommended.
- Chlorhexidine is not recommended for prevention of oral mucositis in patients with solid tumours of the head and neck and who are undergoing radiotherapy.
- Antimicrobial lozenges are not recommended for prevention of radiation-induced oral mucositis.
- Buccolingual guards, using hydroplastic material, can be easily oriented and adapted to an existing radiation stent, adding positional stability and patient comfort; with adequate thickness of material used, the guard can attenuate forward and back scatter radiation, separate the adjacent tissues from metal restorations, and protect the oral mucosa from localized incidents of mucositis [39].

The group of experts agreed that the MASCC guidelines in general terms well address the management of mucositis in patients receiving radiotherapy. These are accepted and used in routine practice by all physicians, and they offered a few recommendations to add to the guidelines in practice. In the absence of any identified pathophysiological differences between mucositis caused by cetuximab/RT and that with radiation alone or CCRT, the management would essentially remain the same. The experts highlighted that symptom control is of utmost importance in the management of mucositis, irrespective of the grade. For example, adding oral opioids for pain control in addition to local anaesthetic agents such as lignocaine and xylocaine.

Since cetuximab does not appear to cause a significant increase in mucositis occurrence compared with radiation alone, it may be feasible to resume cetuximab administration in cases of > grade 3 mucositis [3], as soon as the situation is clinically under control. However, in grade 4 mucositis cetuximab should be stopped, since at that stage mucositis seems to be clinically even more critical than radiation dermatitis. Radiation dosage should not be compromised in such events, unless the infection is of a very severe category or there is a grade 4 reaction that cannot be controlled by symptomatic medications without or with discontinuation of cetuximab and in case of serious systemic infections.

In addition, patients may also be advised to follow some simple daily habits that could reduce the discomfort caused by mucositis, as follows [26]:

- Patients are encouraged to sit upright at a 90° angle and lean their head slightly forward.
- Eat slowly. Food should be cut into small pieces and chewed completely.
- Eat small meals at frequent intervals instead of heavy meals.
- Food taken should be warm, or at room temperature. Hot food and drinks should be avoided. Similarly, crunchy foods such as potato chips and nuts should also be avoided.
- Soft food is always encouraged. Finely chopped cooked meat, fruits, and vegetables should be taken. Patients can also try commercial baby foods, which are nutritious, convenient, and very easy to swallow. Milkshakes that are very high in proteins can also be tried.
- Usage of straws will not only make drinking easy but will also avoid direct contact with the affected portion of the mouth.
- Do not talk while food is in the mouth.
- Acidic foods such as tomatoes, grapes, apple fruits or juices, alcohol and tobacco, and spicy foods should be avoided.
- To relieve the discomfort of dry mouth, patients are asked to rinse mouth with water before and after every meal.

## Conclusions

With newer and emerging therapy options in the management of HNSCC, it is critical that treating physicians are well aware of and updated on the assessment of patient-, tumour-, treatment- and disease-related factors, not just for selecting the most efficacious forms of treatment but also the risk and beneficial aspects of these modalities and agents. However, this should not discourage or dissuade physicians from adopting new forms of therapy, but instead motivate them to better understand the pathophysiology and underlying mechanisms in action for every intervention or treatment approach. The above discussions and recommendations by international head and neck cancer treatment experts were based on literature surveys and experience gained in clinical practice. The recommendations derived from the expert consensus meeting will help to adapt guidelines published in the literature or text books into bedside practice. These recommendations may also serve as a starting point for developing individual institutional side-effect management protocols with adequate training and education in the Asia–Pacific region.
